# Peptide translocation across MOMP, the major outer membrane channel from *Campylobacter jejuni*

**DOI:** 10.1016/j.bbrep.2017.06.007

**Published:** 2017-06-23

**Authors:** Naresh Niranjan Dhanasekar, Soumeya Aliouane, Mathias Winterhalter, Jean-Marie Pagès, Jean-Michel Bolla

**Affiliations:** a Department of Life Sciences and Chemistry, Jacobs University Bremen, 28719 Bremen, Germany; b Aix Marseille University, IRBA, TMCD2, Marseille, France

**Keywords:** *Campylobacter jejuni*, Lipid bilayer, MOMP, Residence time, Single-channel

## Abstract

Here we report on translocation of short poly-arginines across the MOMP porin, the major outer membrane protein in the cell wall of *Campylobacter jejuni*. MOMP was purified to homogeneity from a pathogenic strain of *C. jejuni*. Its reconstitution in lipid membranes and measuring the ion-current revealed two main distinct populations of protein channels which we interpreted as mono and trimers. Addition of poly-arginines causes concentration and voltage dependent ion-current fluctuations. Increasing the transmembrane potential decreases the residence time of the peptide inside the channel indicating successful translocation. We conclude that poly-arginines can cross the outer membrane of *Campylobacter* through the MOMP channel.

## Highlights

• Translocation of short poly-arginines across the MOMP channel has been determined.
• Penta-arginine and Hepta-arginine translocate across the MOMP channel.
• Voltage dependent kinetics to distinguish binding from translocation.

## 1 Introduction

*Gram-negative* bacteria have a complex outer membrane which plays an important role in host-pathogen interaction. *Campylobacter jejuni*, a major cause of food-borne infections worldwide, raised our interest as this bacteria adapts its transcriptome in response to variation of different conditions such as temperature, pH and ionic composition [1], [2], [3]. In *Campylobacter*, to date, three channel forming proteins have been identified namely, the major outer membrane protein (MOMP), Omp50 and CadF [2], [4], [5], [6]. MOMP (or PorA) is present in *C. jejuni* (mainly found in chicken which have a body temperature of 42 °C), *C. coli* (mainly found in pigs which have a body temperature of 37 °C) and *C. lari* (mainly found in wild birds which have a body temperature of 42 °C). Particularly, it has been shown that MOMP is regulated as a function of temperature and pH of the growth media indicating a key role of porins in bacteria adaptation to environment [2], [3].

Previously, we and others reported biochemical properties of MOMP, however, little is known about the transport properties of small molecules across the MOMP protein [7], [8], [9]. For example, liposome swelling assays showed the uptake of several antibiotics through this porin [9], [10]. In contrast, most permeation studies have been done in porins from *E. coli*, for instance, OmpF that allows the passive diffusion of small water soluble molecules with an exclusion limit of approximately 600 Da [11]. Sugar specific channels like maltoporins have an affinity site facilitating the uptake of carbohydrates through specific porins [12], [13], [14], [15]. Similar transport studies have been performed on polypeptides [16], [17]. Moreover, the principle of selective uptake by biological protein channel found a number of biotechnological applications, its most prominent being DNA sequencing [18], [19], [20], [21], [22], [23], [24]. Recently we have shown that transmembrane potential and the peptide length modify charged peptide translocation across the protein pore [16]. Increasing the transmembrane voltage pulls the peptide stronger, and thus, the peptides should translocate faster reflected by a reduced channel blockage (or in other words the peptide residence time). While the mitochondrial presequence peptide, pF_1_β showed a reduced residence time in TOM40 [16], poly-arginine required higher voltages to show voltage dependent reduction in translocation across *Nocardia farcinica* [25]. In contrast, poly-arginine apparently is unable to translocate through OmpF from *E. coli* [26].

In the present study, we isolated and purified MOMP from *C. jejuni.* For transport studies we reconstituted single detergent solubilised MOMP into artificial lipid bilayers. Subsequently, we investigated the translocation of short range cationic peptides by measuring the time dependent ion-current fluctuations as a function of peptide concentration and applied transmembrane potential.

## 2 Materials and methods

### 2.1 Bacterial strain

*C. jejuni* 85 H was cultivated on Columbia agar (GC) plates (Oxoid, Dardilly, France) during 48 h at 42 °C under microaerophilic conditions obtained by CampyGen compact (Oxoid).

### 2.2 Chemicals and peptides

Potassium chloride, 2-(*N*-morpholino) ethanesulfonic acid (MES), n-pentane and hexadecane are from Applichem (Darmstadt, Germany). All the chemicals were of analytical grade with > 95% purity. 1, 2-diphytanoyl-*sn*-glycero-3-phosphatidyl choline (DPhPC, Avanti Polar Lipids, Alabaster, AL, USA). Milli-Q water was used throughout the experiments. In the present study we used Tri-arginine (H-RRR-OH acetate salt, M_w_: 0.486 kDa), Penta-arginine (H-RRRRR-OH trifluoroacetate salt, M_w_: 0.798 kDa) and Hepta-arginine (H-RRRRRRR-OH trifluoro acetate salt, M_w_: 1.111 kDa) from Bachem AG (Bubendorf, Switzerland).

### 2.3 *Campylobacter* porin purification

MOMP purification was performed from the strain 85H as previously described [27]. The protein concentrations were determined using the BCA (Bicinchoninic acid) protein assay (Pierce BCA protein assay kit, Thermoscientific, Dardilly, France), using bovine serum albumin as standard. The protein was kept at 20 °C until use.

### 2.4 SDS-PAGE and immunoblotting

Analyses of protein by 10% SDS-Polyacrylamide Gel Electrophoresis (SDS-PAGE) and immunoblotting using a specific rabbit antiserum were performed as previously described [2].

### 2.5 Single-channel conductance measurements

**Fig. 1:**
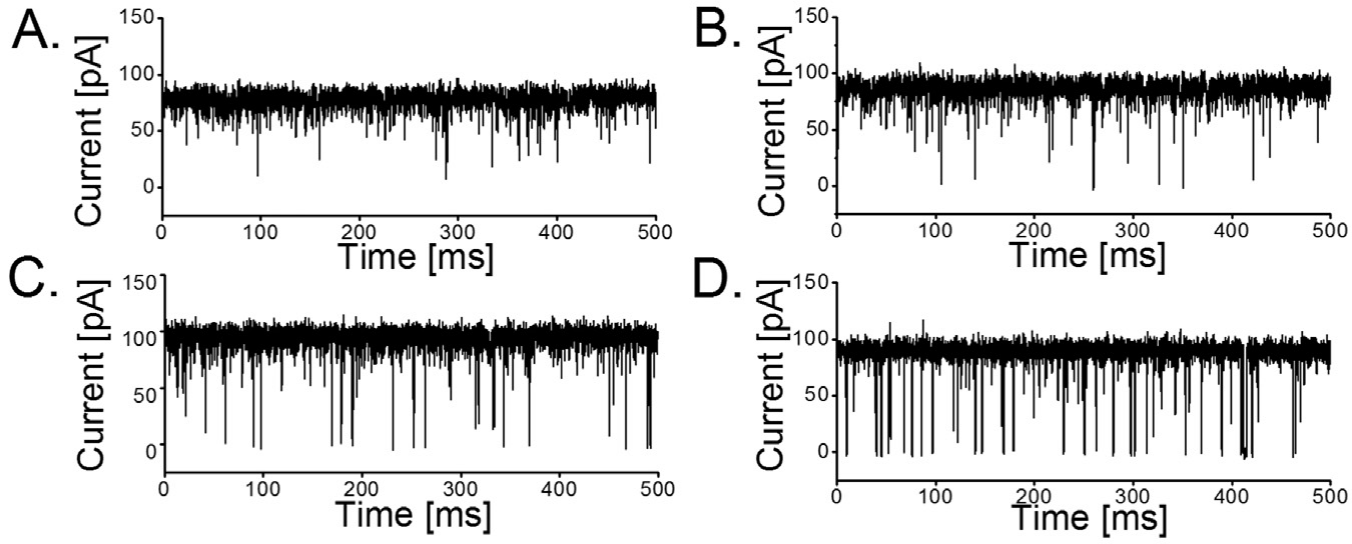
Typical ion-conductance traces of monomeric MOMP in presence of poly-arginines at +125 mV. **A.** In the absence of peptides; **B.** 3 µM of Tri-arginine; **C.** 3 µM of Penta-arginine; and **D.** 3 µM of Hepta-arginine. Experimental conditions: 1 M KCl, 10 mM MES, pH 6.0, T= 20 °C. The peptide is added to the *trans* compartment of the cuvette corresponding to the electrode at positive voltage. For the sake of clarity the traces were filtered at 5 kHz. MOMP is added on the *cis* (ground) side (n = 4).

The planar lipid bilayer was formed using solvent free lipid bilayer technique [28], [29]. In brief, the cuvettes used for our bilayer experiments consist of *cis* (electrical ground and side of protein addition if not otherwise indicated) and *trans* chambers separated by a 25 µm thick Teflon film (Goodfellow, Cambridge, UK) carrying an aperture with a diameter of 40–70 µm. Prior to forming a lipid bilayer, the aperture is pre-painted with 1 μl of 1% hexadexane in hexane. 5% solution of DPhPC in hexane is commonly used as lipid stock. The chambers are filled with electrolyte solution which typically consists of 1 M KCl, 10 mM MES, pH 6.0 with a total solution volume of 2.5 ml. On top of the aqueous phase about 1 μl of the lipid stock solution is placed. After 10 min allowing for evaporation of solvent, the membrane is formed by raising and lowering the water level. To investigate the outer membrane protein of *C. jejuni*, MOMP was added to the *cis* (ground) side of the chamber to a final concentration of 5 ng/ml and the channel insertion was facilitated by rapid mixing of the content of the chamber while applying a transmembrane potential of −199 mV. Electrical recordings were made through a pair of Ag/AgCl electrodes (World Precision Instruments, Sarasota, FL, USA), attached to an Axon Instruments 200B amplifier (Axon Instruments Inc., Sunnyvale, CA, USA) in the voltage clamp mode. Data were filtered by a low pass Bessel filter at 10 kHz and directly saved into the computer memory with a sampling frequency of 50 kHz. Data analysis was performed using Clampfit software of version 10.4 (Axon Instruments Inc., Sunnyvale, CA, USA). To obtain quantitative information on the interaction of peptides with the channel we assume that peptide binding or penetration to the channel cause sufficiently strong reduction of the ion-current compared to the typical background noise. Comparing Fig. 1**A** in absence of any peptides with Fig. 1**B** with low amount of short peptides reveals noticeable extra noise. This is even more pronounced for longer peptides (Fig. 1**C** and **D**). To give a quantitative description we suggest a simple model assuming binding of the substrate, in this case the peptides in solution, to the inside of the channel, [30]:

$$open\;channel+peptide\overset{k_{on}}{\underset{k_{of}}{<=>}}closed\;channel$$

For further analysis we should note that in our experiment we work under dilute concentrations and thus, the number of entries is in good approximation proportional to the concentration of the peptides in solution. The on-rate *k*_*on*_ (association rate) is obtained by counting the number of ion-current blockage events per time n [s^−1^] divided by the concentration [c] of the peptide [13], [26], [29], [30]. Furthermore, in some cases we have trimeric MOMP and the ion-current blockages may originate independently from one of the three monomers.

$$k_{on}=n/3\left[c\right]$$

On the other hand the opening is a statistical event and correlated to the strength of the binding. A channel which is closed at t = 0 will have the probability R(t) to open at time t. Within the above described simple binding model an exponential function R(t) = e^-t/τ^. Fitting the life-time distribution of a closed channel by an exponential parameter τ (or dwell time) yields the off-rate (dissociation rate) *k*_*off*_ = 1/τ [30]. The equilibrium binding constant *K*_*a*_ [M^−1^] of the peptide to the channel is given by the ratio of the rates *K*_*a*_ = *k*_*on*_/*k*_*off*_.

## 3 Results

MOMP was purified as outlined in the Section 2 (Fig. S1). A single MOMP channel when reconstituted into a planar lipid bilayers showed a monomer or trimeric channel conductance of 0.7 ± 0.2 nS and 2.2 ± 0.2 nS in 1 M KCl buffered with MES 10 mM at pH 6.0 [27]. At positive voltages, the channel is more silent, on the other hand the channel has long upward spikes at negative voltages (*cis* (ground) side protein addition). We use this feature to attribute an orientation of the inserted protein. Additionally we observed in a few cases also a conductance of 1.3 ± 0.2 nS which we may attribute to the rare events of dimer insertion (Fig. S2**A** and **B**). Fig. S2**C**, shows the gating of a trimeric MOMP channel visualized in a three-step fashion. The threshold potential inducing the trimeric or the monomeric pore gating was found to be > + 175 mV, while the channels were not affected by gating up to + 150 mV and hence, were suitable for our investigation on peptide translocation studies (Fig. S2**D**). To investigate the effect of poly-arginines, we reconstituted single MOMP monomer into planar lipid bilayer and measured the ion-current fluctuations in the presence and absence of poly-arginine peptides. Fig. 1 represents the ion-current traces of the interaction of the various cationic peptides with the single monomeric MOMP channel. At positive voltage, in the absence of the peptides, ion-current fluctuations were stable without any modification in the conductance of the MOMP channel (Fig. 1**A**), while addition of 3 µM concentrations of these cationic peptides to the *trans* side of the bilayer induced reversible monomer blockages of MOMP (Fig. 1**B, C and D**). Tri-arginine (3 µM) produced visible but very short and not fully resolvable monomer blockages (Fig. 1**B**). Subsequently, we used Penta-arginine and Hepta-arginine, and found that both produced complete monomer blockage (Fig. 1**C and D**). These blockages reduced the average conductance in a peptide size dependent manner. The addition of cationic peptide to the *cis* (ground) side of the chamber required *trans*-negative potential to promote the peptide entry into MOMP. To test the orientation effect, we added the protein on the *trans* side and performed similar measurements only with Hepta- and Pentarginine with a concentration ranging from 1 to 10 µM. Addition of protein on the *trans* side resulted in reverse orientation of MOMP (flickering on positive voltage), where the ion-current trace is decorated with downward spikes at positive voltages, with a slightly lower conductance of 0.5 ± 0.1 nS at + 100 mV (Fig. S3).

**Fig. 2:**
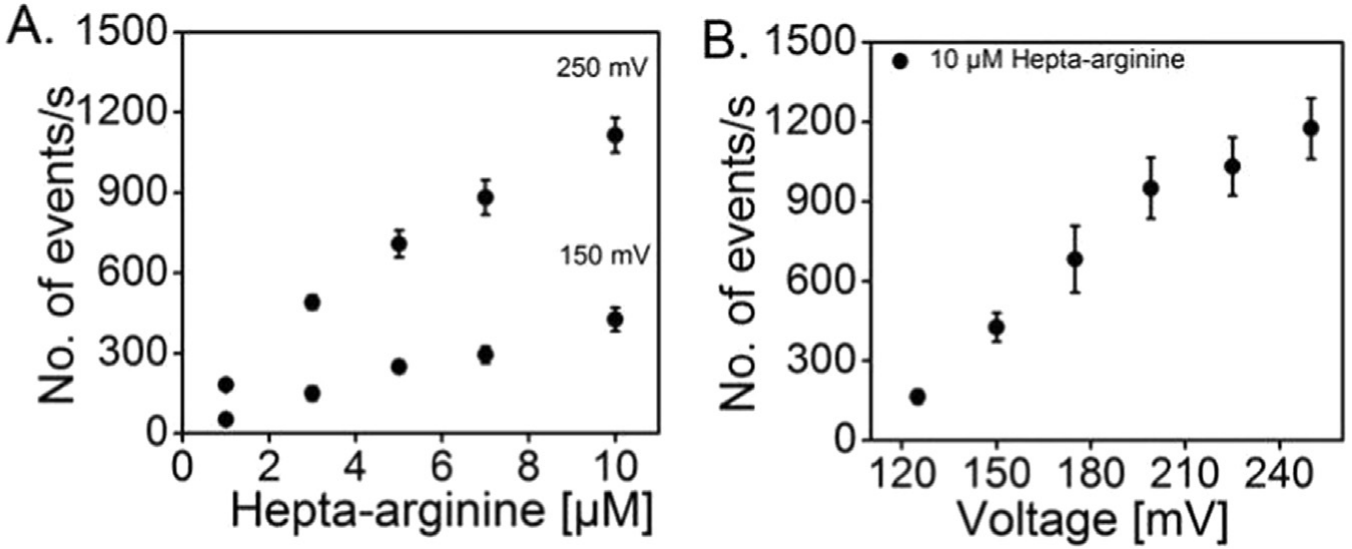
Analysis of the ion-current fluctuations. **A.** Hepta-arginine concentration dependence of binding events; data were analysed at applied voltages, +150 and +250 mV; and **B.** Frequency of binding events of the peptide (10 µM) vs. applied transmembrane potential. Experimental conditions: 1 M KCl, 10 mM MES, pH 6.0, T= 20 °C. MOMP is added on the *cis* (ground) side (n = 4).

We further analysed the ion-current blockages induced by polyarginines under various conditions. We observed that, whatever the applied voltage, the number of binding events increased with the concentration of the peptide, Fig. 2**A** shows the binding events for Hepta-arginine from 1 to 10 µM. The frequency of binding events also increased with the increase of the applied transmembrane potential from +125 to +250 mV (Fig. 2**B**).

**Fig. 3:**
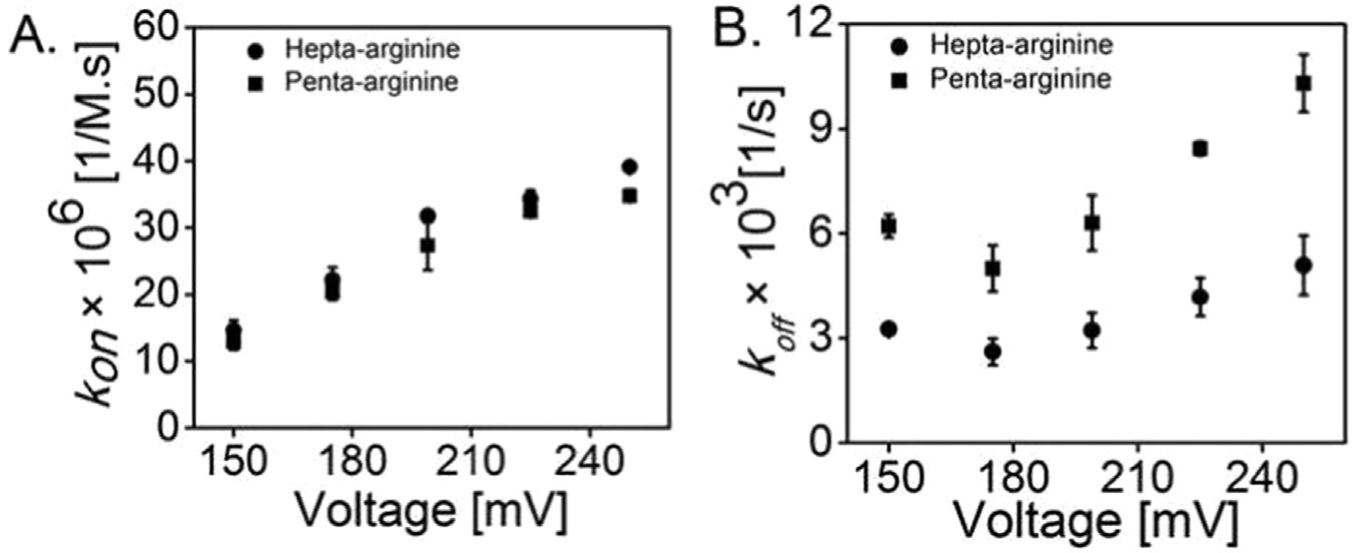
Kinetic rates of Hepta- and Penta-arginine binding to single monomers of MOMP. **A.** Voltage dependence of the on-rate; and **B.** off-rate. Experimental conditions: 1 M KCl, 10 mM MES, pH 6.0, T= 20 °C and 10 µM of final peptide concentration was added to the *trans* side of the bilayer chamber. MOMP is added on the *cis* (ground) side (n = 4).

**Fig. 4:**
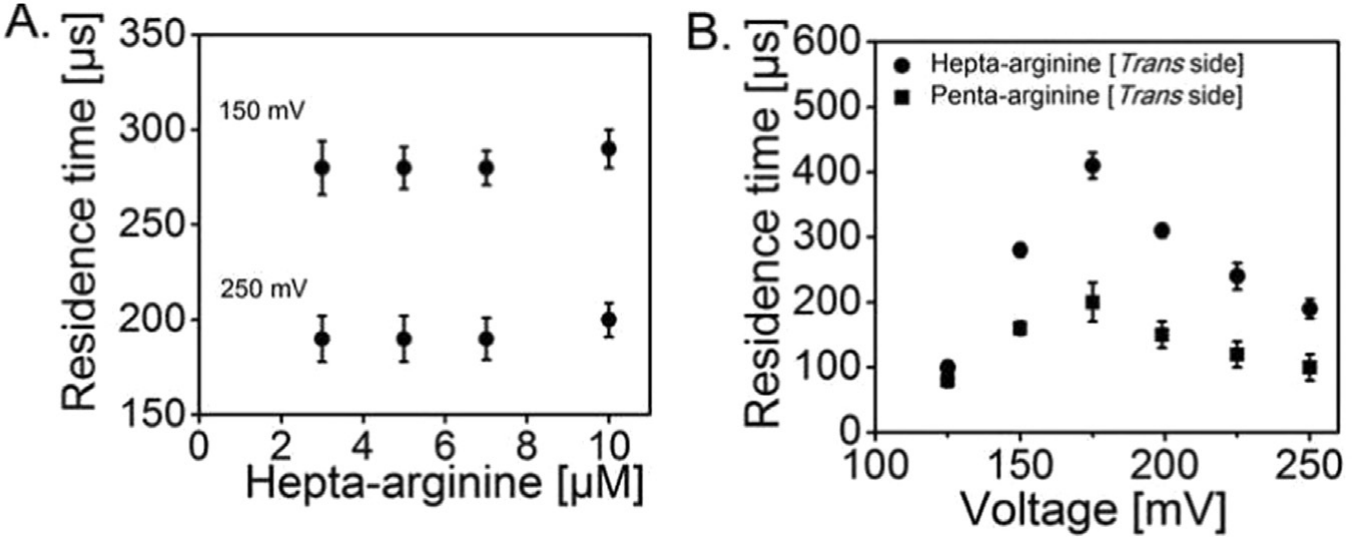
**A.** The residence time as a function of peptide concentration at applied transmembrane potential for Hepta-arginine, +150 and +250 mV; and **B.** as a function of applied transmembrane potential for Hepta- and Penta-arginine from +125 to +250 mV. Experimental conditions: 1 M KCl, 10 mM MES, pH 6.0, T= 20 °C. 10 µM concentration of the peptide was used with an applied transmembrane potential from +125 to +250 mV for **A**. MOMP is added on the *cis* (ground) side (n = 4).

The ion-current traces can be analysed as outlined in the Methods section. Fig. 3**A** shows the association rate *k*_*on*_ for Hepta-arginine and Penta-arginine as a function of applied transmembrane potential. Both the Penta-arginine and Hepta-arginine exhibited almost identical increasing values for applied transmembrane potentials from + 150 to +250 mV (Fig. 3**A**). For instance, the on-rate at +150 mV was in the range of 13 ± 2 10^6^ M^−1^ s^−1^ for Penta- and Hepta-arginine (Table 1). In Fig. 3**B,** we show the dissociation rates *k*_*off*_ revealing about twice higher off-rates for the smaller penta-arginines. As expected in the dilute regime the residence times of the peptides in the channel should be independent of the concentration and shorter for higher voltages (Fig. 4**A**). For Penta- and Hepta-arginine (Fig. 4**B),** the average residence time of the peptide in the channel first increased with applied transmembrane potential up to +175 mV from ~100 to 410 µs. Surprisingly, further increase of the voltage caused a reduction of one-fold in the residence time, i.e., from ~ 410 to 190 µs, indicating a translocation of this peptide. For tri-arginine we could visualize the number of events (Fig. 1**B and** S4**A**) but not resolve the residence time sufficiently, the residence time was always in the range of ~70 ± 5 µs (Fig. S4**B**) corresponding to the time resolution of the instrument. Control measurements by adding the peptide (Hepta- and Penta-arginine) to the *cis* (ground side) of the Teflon chamber did not reveal significant differences both in the residence time and the frequency of binding events for Hepta- and Penta-arginine, respectively (Fig. S5). For instance, decrease in the residence time for the successful translocation of Hepta-arginine was from 410 to 200 µs which is almost similar, when the peptide was added on the *trans* side.

**Table 1:** Association (*k*_*on*_), dissociation (*k*_*off*_) and binding (*K*_*a*_) constants of the interaction between the peptides and the MOMP channel.

**Penta-arginine**				**Hepta-arginine**			
**Voltage (mV)**	***k***_***on***_**(M**^**−1**^** s**^**−1**^**) × 10**^**6**^	***k***_***off***_**(s**^**−1**^**) × 10**^**3**^	***K***_***a***_**(M**^**−1**^**) × 10**^**3**^	**Voltage (mV)**	***k***_***on***_**(M**^**−1**^** s**^**−1**^**) × 10**^**6**^	***k***_***off***_**(s**^**−1**^**) × 10**^**3**^	***K***_***a***_**(M**^**−1**^**) × 10**^**3**^
+150	12.9 ± 1.2	6.2 ± 0.3	2.0 ± 0.5	+ 150	14.5 ± 1.5	3.2 ± 0.1	4.4 ± 0.3
+175	20.4 ± 1.2	5.0 ± 0.6	4.1 ± 0.3	+ 175	22.2 ± 0.8	2.6 ± 0.3	8.5 ± 0.2
+250	34.7 ± 0.8	10.3 ± 0.8	3.3 ± 0.2	+ 250	39.1 ± 0.5	5.0 ± 0.8	7.7 ± 0.3

## 4 Discussion

Most studies that address the permeation of solutes (including small antibiotics) through MOMP channel were based on *in-vitro* transport studies which utilize liposome swelling assays. These studies allowed the identification of numerous substrates permeation through the aqueous route (MOMP channel) [9]. Here, we shed direct insights in the permeation of short ranged poly-arginines into the MOMP channel under single molecule level.

The MOMP protein was isolated after selective extraction of proteins from outer membrane vesicles following ion exchange chromatography [7]. We previously showed that the MOMP is able to induce pores in artificial lipid membranes [8]. Our reconstitution protocol of 1% Octyl-POE solubilized MOMP into solvent free membranes resulted in two main channel conductance values of 0.7 ± 0.2 nS (monomer) and 2.2 ± 0.2 nS (trimer) in 1 M KCl, 10 mM MES, pH 6.0. Interestingly, the conductance of the monomer was slightly reduced when the protein was added to the *trans* side of the bilayer chamber (0.5 nS, + 100 mV). In addition, to mono- and trimers, we rarely observe a dimer-like pattern having an average conductance of about 1.3 ± 0.2 nS. Zhuang and his co-workers argued that this might corresponds to the observation of protein band at ~ 70 kDa, which was attributed to the formation of a pseudo-dimer or an unfolded monomer [31]. Dimers were found to act as intermediate in trimerization. Similar, studies have been reported with the mesoscopic porin OmpF [32]. It has been shown that, each subunit of the OmpF trimers forms distinct membrane channels in the artificial membranes. The dimers formed by MOMP are symmetric in nature (G = 1.20 ± 0.2 nS, − 100 mV), in contrast, asymmetric dimers are formed by OmpF channels.

Binding and translocation can be distinguished using the voltage dependency of the association and disassociation rates [25]. If the time spent by a charged molecule inside the channel increases with increasing voltage, we conclude that the molecule is pulled inside on one side of the channel without being translocated. On the other hand, if the residence time decreases with increasing voltage we conclude that the molecule is able to follow the electric field gradient leaving faster to the other side. In the case of Hepta-arginine, the residence time increased from ~100 to 410 µs with increasing in the applied voltage from +125 to +175 mV. This indicates that the peptide binds to channel without being translocated. Increasing the voltage from +175 to +250 mV we observed about a one-fold reduction in the residence time, i.e., from ~410 to 190 µs which signifies a successful translocation of the peptide (Fig. 4**)**. Similarly, Penta-arginine permeated faster with increasing voltages and the threshold potential for successful translocation to pull the peptide out of the channel remains the same as +175 mV for both Penta- and Hepta-arginine. In contrast to MOMP, OmpF channel behaves differently, in this case increasing the applied voltage induces an increase of the residence time [26]. On the other hand, a strong difference in the voltage takes place in *Nocardia* channel for pulling Hepta-arginine (> −150 mV) and Penta-arginine (> –175 mV) [25]. Reversing the external voltage and the side of protein and peptide addition revealed the asymmetry of the channel. For instance, addition of Hepta-arginine to the *cis* (ground) side (protein addition on *trans* side) of the bilayer chamber produced strong monomer blockages with negative voltages. Similar to *Nocardia* the strength of the channel–peptide interaction is in the order of Hepta-arginine > Penta-arginine > Tri-arginine.

Translocation of biomolecules across the bio-membranes is an ideal process. Indeed, direct evidence for the translocation of uncharged compounds is not possible [33]. In contrast, the peptide length and applied voltage is critical for the successful translocation of the charged polypeptide across several biological nanopores [25].

## 5 Conclusion

In the present study, MOMP has been characterized at a single molecular level. Using the voltage dependence of the ion-current fluctuations we conclude that small poly-arginine peptides may permeate through MOMP. We could resolve the residence time of hepta and penta-arginine whereas smaller peptide was below the time resolution. Under natural condition in absence of membrane potentials, arginine as a possible substrate for *Campylobacter* may permeate by diffusion.

## Footnotes

Appendix B Supplementary data associated with this article can be found in the online version at doi:10.1016/j.bbrep.2017.06.007.

## References

[1] Biswas D., Fernando U.M., Reiman C.D., Willson P.J., Townsend G.G., Potter A.A., Allan B.J. (2007). Correlation between in vitro secretion of virulence-associated proteins of *Campylobacter jejuni* and colonization of chicken. Curr. Microbiol..

[2] Dedieu L., Pagès J.-M., Bolla J.M. (2002). Environmental regulation of *Campylobacter jejuni* major outer membrane protein expression in *Escherichia coli* monitored by using Green Fluorescent protein. Appl. Environ. Microbiol..

[3] Dedieu L., Pagès J.-M., Bolla J.M. (2008). The omp50 gene is transcriptionally controlled by a temperature-dependent mechanism conserved among thermophilic *Campylobacter* species. Res. Microbiol..

[4] Bolla J.M., Dé E., Dorez A., Pagés J.-M. (2000). Purification, characterization and sequence analysis of Omp50, a new porin isolated from *Campylobacter jejuni*. Biochem. J..

[5] Mamelli L., Pagès J.-M., Konkel M.E., Bolla J.M. (2006). Expression and purification of native and truncated forms of CadF, an outer membrane protein of *Campylobacter*. Int. J. Biol. Macromol..

[6] Mamelli L., Dedieu L., Dé E., Konkel M.E., Pagès J.-M., Bolla J.M. (2007). Chromosomal His-tagging: an alternative approach to membrane protein purification. Proteomics.

[7] Bolla J.M., Loret E., Zalewski M., Pagés J.-M. (1995). Conformational analysis of the *Campylobacter jejuni* porin. J. Bacteriol..

[8] Dé E., Jullien M., Labesse G., Pagés J.-M., Molle G., Bolla J.M. (2000). MOMP (major outer membrane protein) of Campylobacter jejuni; a versatile pore-forming protein. FEBS. Lett..

[9] Page W.J., Huyer G., Huyer M., Worobec E. (1989). Characterization of the porins of *Campylobacter jejuni* and *Campylobacter coli* and implications for antibiotic susceptibility. Antimicrob. Agents Chemother..

[10] Iovine N.M. (2013). Resistance mechanisms in *Campylobacter jejuni*. Virulence.

[11] Nikaido H., Rosenberg E.Y. (1983). Porin channels in *Escherichia coli*: studies with liposomes reconstituted from purified protein. J. Bacteriol..

[12] Benz R., Schmid A., Vos-Scheperkeuter G.H. (1987). Mechanism of sugar transport through the sugar specific LamB channel of *Escherichia coli*. J. Membr. Biol..

[13] Danelon C., Brando T., Winterhalter M. (2003). Probing the orientation of reconstituted maltoporin at single protein level. J. Biol. Chem..

[14] Biswas S., Mohammad M.M., Patel D.R., Movileanu L., van den Berg B. (2007). Structural insight into OprD substrate specificity. Nat. Struct. Mol. Biol..

[15] van den Berg B., Prathyusha Bhamidimarri S., Dahyabhai Prajapati J., Kleinekathöfer U., Winterhalter M. (2015). Outer-membrane translocation of bulky small molecules by passive diffusion. Proc. Natl. Acad. USA.

[16] Mahendran K.R., Romero-Ruiz M., Schlosinger A., Winterhalter M., Nussberger S. (2012). Protein translocation through Tom40: kinetics of peptide release. Biophys. J..

[17] Harsman A., Niemann M., Pusnik M., Schmidt O., Burmann B.M., Hiller S., Meisinger C., Schneider A., Wagner R. (2012). Bacterial origin of a mitochondrial outer membrane protein translocase. J. Biol. Chem..

[18] Pastoriza-Gallego M., Breton M.F., Discala F., Auvray L., Betton J.M., Pelta J. (2014). Evidence of unfolded protein translocation through a protein nanopore. ACS Nano.

[19] Akeson M., Branton D., Kasianowicz J.J., Brandin E., Deamer D.W. (1999). Microsecond time-scale discrimination among polycytidylic acid, polyadenylic acid, and polyuridylic acid as homopolymers or as segments within single RNA molecules. Biophys. J..

[20] Payet L., Martinho M., Merstorf C., Pastoriza-Gallego M., Pelta J., Viasnoff V., Auvray L., Muthukumar M., Mathé J. (2015). Temperature effect on ionic current and ssDNA transport through nanopores. Biophys. J..

[21] Movileanu L., Schmittschmitt J.P., Scholtz J.M., Bayley H. (2005). Interactions of peptides with a protein pore. Biophys. J..

[22] Kakish J., Lee D., Lee J.S. (2015). Drugs that binds to alpha-synuclein. ACS Chem. Neurosci..

[23] Mayer M., Yang J J. (2013). Engineering ion channels as emerging tools for chemical biology. Acc. Chem. Res..

[24] Kasianowicz J.J., Balijepalli A.K., Ettedgui J., Forstater J.H., Wang H.Y., Zhang H.S., Robertson J.W.F. (2016). Analytical applications of pore-forming proteins. Biochim. Biophys. Acta.

[25] Singh P.R., Bárcena-Uribarri I., Modi N., Kleinekathöfer U., Benz R., Winterhalter M., Mahendran K.R. (2012). Pulling peptides across nanochannels: resolving peptide binding and translocation through the hetero-oligomeric channel from *Nocardia farcinica*. ACS Nano.

[26] Lamichhane U., Tuhidul I., Prasad S., Weingart H., Mahendran K.R., Winterhalter M. (2013). Peptide translocation through the mesoscopic channel: binding kinetics at the single molecule level. Eur. Biophys. J..

[27] Ferrara G.M.L., Wallat G.D., Moynié L., Dhanasekar N.N., Aliouane S., Acosta-Gutiérrez S., Pagès J.-M., Bolla J.-M., Winterhalter M., Ceccarelli M., Naismith J.H. (2016). MOMP from *Campylobacter jejuni* is a trimer of 18 stranded β–barrel monomers with a Ca^2+^ ion bound at the constriction zone. J. Mol. Biol..

[28] Montal M., Mueller P. (1972). Formation of biomolecular membranes from lipid monolayers and a study of their electrical properties. Proc. Natl. Acad. Sci. USA.

[29] Gutsmann T., Heimburg T., Keyser U., Mahendran K.R., Winterhalter M. (2015). Protein reconstitution into freestanding planar lipid membranes for electrophysiological characterization. Nat. Protoc..

[30] Colquhoun D., Hawkes A.G., Sackmann B., Neher E. (1995). The principle of the stochastic interpretation of ion-channel mechanisms. Single Channel Recording.

[31] Zhuang J., Engel A., Pagés J.-M., Bolla J.M. (1997). The *Campylobacter jejuni* porin trimers pack into different lattice types when reconstituted in the presence of lipid. Eur. J. Biochem..

[32] Visudtiphole V., Thomas M.B., Chalton D.A., Lakey J.H. (2005). Refolding of *Escherichia coli* outer membrane protein F in detergent creates LPS-free trimers and asymmetric dimers. Biochem. J..

[33] Mahendran K.R., Hajjar E., Mach T., Lovelle M., Sousa I., Kumar A., Spiga E., Weingart H., Gameiro P., Winterhalter M. (2010). Molecular basis of enrofloxacin translocation through a bacterial porin – when binding does not imply translocation. J. Phys. Chem. B.

