# Supplementary material for: Peptide translocation across MOMP, the major outer membrane channel from Campylobacter jejuni
Source: Biochem Biophys Rep. 2017 Jun 23;11:79–83. doi: 10.1016/j.bbrep.2017.06.007 (PMC5614690; doi:10.1016/j.bbrep.2017.06.007)
Supplement: Supplementary file 2 — Supplementary material [file mmc2.doc]

**Supplementary Figures and Legends**


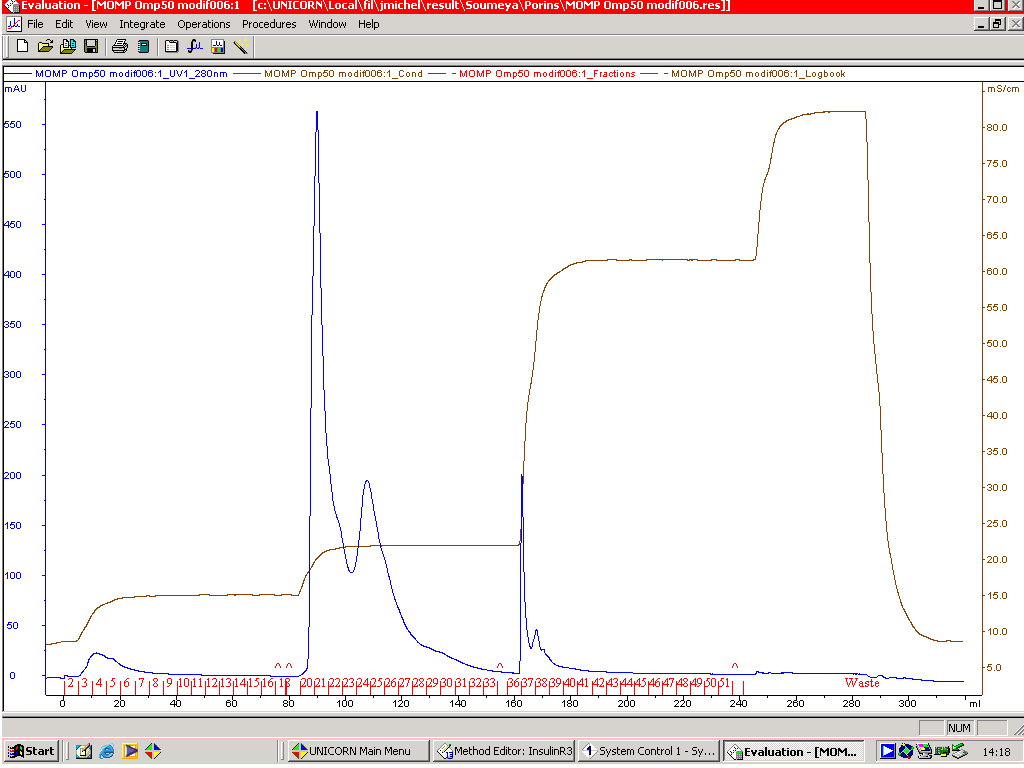


3

2

OD 280nm

Conductimetry


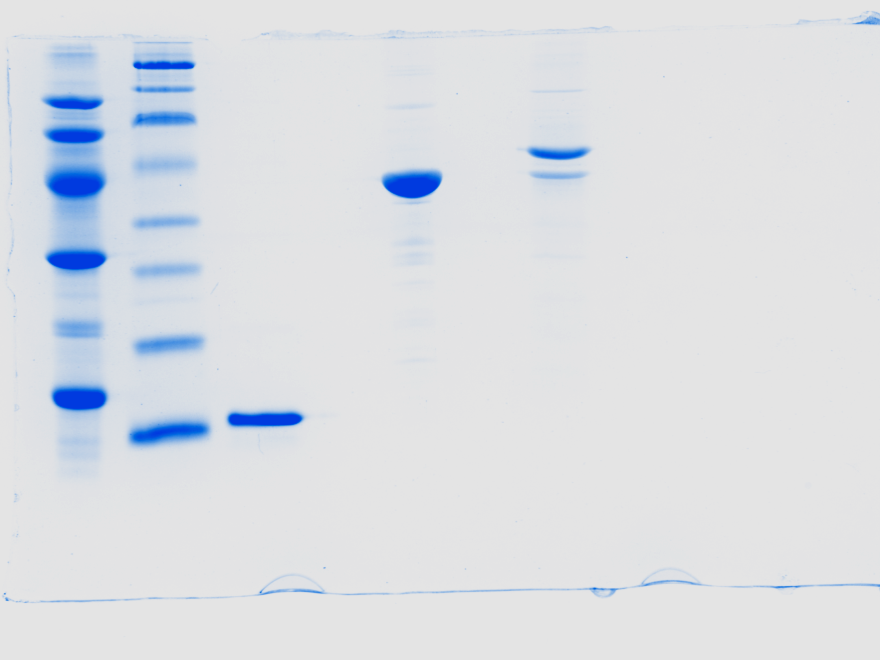


66

97

45

20

30

14.4

MOMP

kDa

A

4

1

**Figure S1. MOMP purification.** **A.** Ion exchange chromatography of outer membrane proteins from *C. jejuni* 85H. 30 ml of Octyl-POE extract was loaded onto the Mono-Q column. The OD (Optical density) 280 is indicated on the left axis and the conductimetry is indicated on the right axis. Four peaks of protein elution were observed and identified by SDS-PAGE as a protein of 45 kDa in the second (Fractions: 20-23) peak, in the third (Fractions: 24-31) peak and in the fourth (Fractions: 36-39) one. The second, the third and the fourth peak correspond to MOMP; and **B.** 10% SDS-PAGE of purified and concentrated MOMP (9µg) stained with Coomassie Blue.


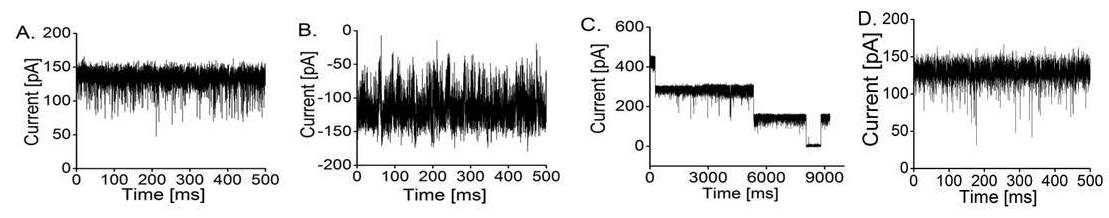


**Figure S2.** Typical ion-current traces of single dimeric form of MOMP at applied transmembrane potential of **A.** +100 mV; **B.** –100 mV; **C.** Gating of the single trimeric MOMP channel in a three-step fashion at an applied voltage of +199 mV; and **D.** Ion-current trace of a single monomeric form of MOMP at applied transmembrane potential of +175 mV. Experimental conditions: 1 M KCl, 10 mM MES, pH 6.0, T= 20°C. MOMP is added on the *cis* (ground) side.


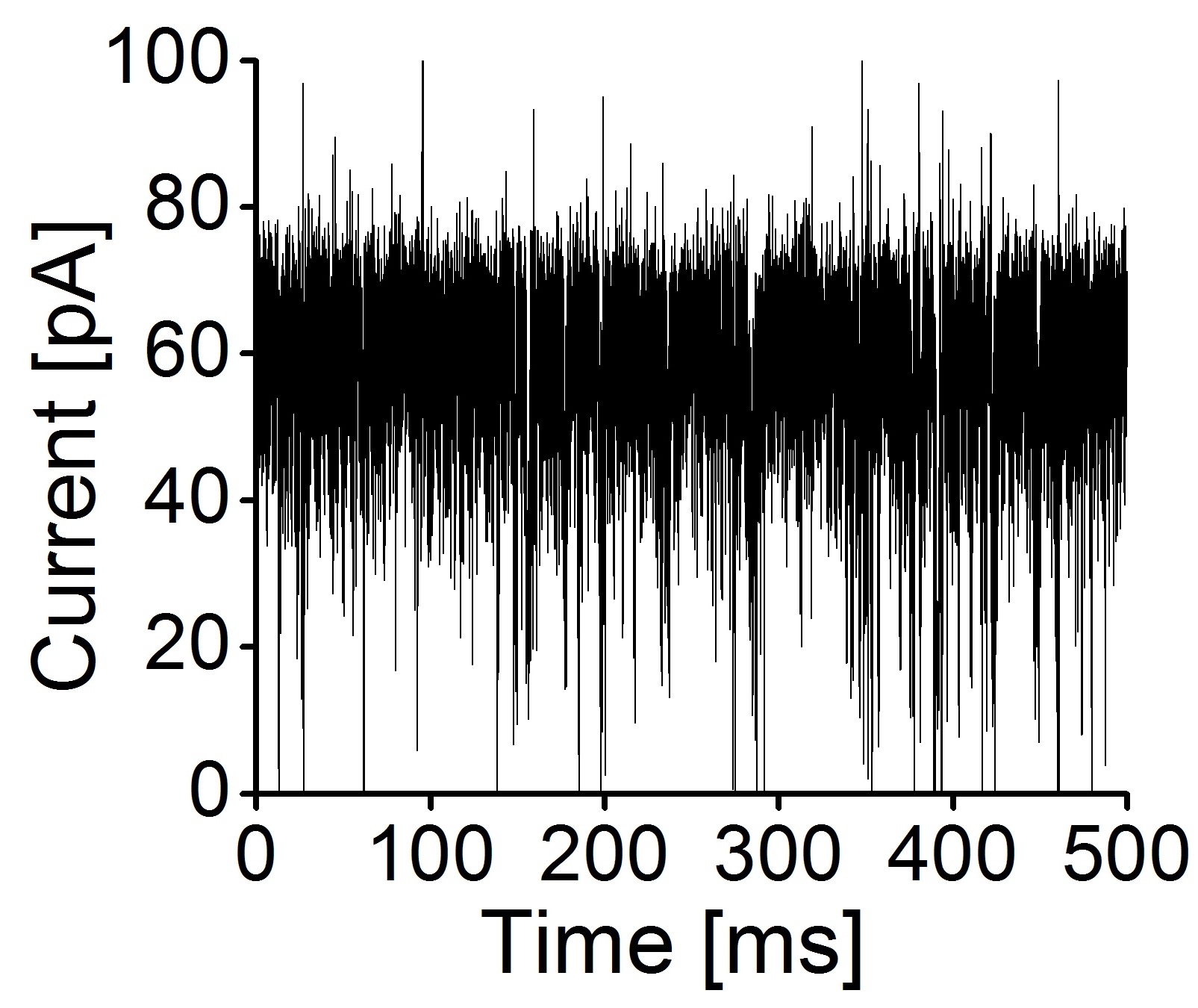


**Figure S3.** Typical ion-current trace of a single MOMP monomeric form at applied transmembrane potential of +100 mV with protein addition on *trans* side (reverse orientation). Experimental conditions: 1 M KCl, 10 mM MES, pH 6.0, T= 20°C.


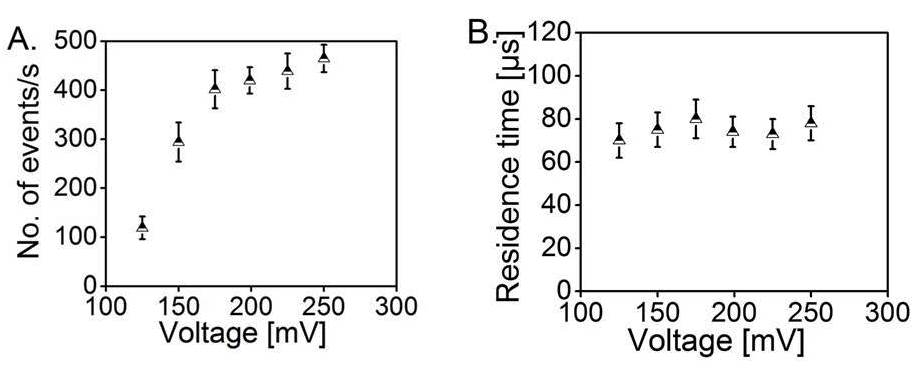


**Figure S4.** Peptide binding analysis for Tri-arginine. **A.** The number of binding events as a function of applied transmembrane potential from +125 to +250 mV; and **B.** The residence time as a function of applied transmembrane potential from +125 to +250 mV. Experimental conditions: 1 M KCl, 10 mM MES, pH 6.0, T= 20°C and 10 µM concentration of Tri-arginine was added on the *trans* side of the bilayer chamber. MOMP is added on the *cis* (ground) side (n = 4).


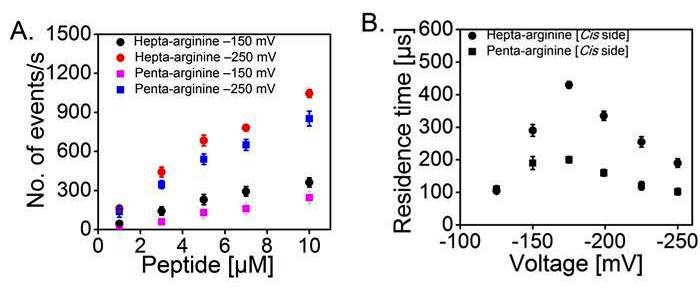


**Figure S5. A.** The number of binding events as a function of peptide concentration at applied transmembrane potential for Hepta- and Penta-arginine, –150 and –250 mV; and **B.** Residence time as a function of applied transmembrane potential for Hepta- and Penta-arginine from –125 to –250 mV. Experimental conditions: 1 M KCl, 10 mM MES, pH 6.0, T= 20°C. MOMP is added on the *trans* and the peptide on the *cis* (ground) side (n =4).
